# Organelle-centered ISG15 biology: distinguishing covalent ISGylation from interferon-associated responses

**DOI:** 10.3389/fimmu.2026.1960492

**Published:** 2026-09-03

**Authors:** Zhuoer Li, Chunli Wang, Jinrong Zhang, Zhiqiang Guo

**Affiliations:** Department of Obstetrics and Gynecology, Shengjing Hospital of China Medical University, Shenyang, Liaoning, China

**Keywords:** autophagy, biomarkers, endoplasmic reticulum stress, ISG15, ISGylation, mitochondrial dynamics, organelle homeostasis

## Abstract

ISGylation is an interferon-inducible ubiquitin-like post-translational modification mediated by the interferon-stimulated gene 15 conjugation system. Initially characterized as an antiviral effector pathway, ISGylation is now increasingly recognized as a regulator of organelle homeostasis and cellular stress responses. This review summarizes emerging evidence linking ISG15-related mechanisms and covalent ISGylation to major organelle systems, including mitochondria, the endoplasmic reticulum–Golgi axis, endolysosomal compartments, ribosome-associated translation, and lipid droplets. We distinguish covalent ISGylation from free ISG15 signaling and ubiquitin specific protease 18-mediated interferon regulation, and further classify existing findings into evidence-based levels ranging from substrate-validated modification to correlative interferon signatures. At the mitochondrial level, direct and pathway-level evidence implicates ISG15 biology in DRP1-mediated fission, MFN1/2-associated mitophagy, oxidative metabolism, and redox regulation; direct effects of MFN1/2 ISGylation on mitochondrial fusion remain unproven. Along the ER–Golgi axis, ISG15-related pathways intersect with unfolded protein response signaling, endoplasmic reticulum-associated degradation, and stimulator of interferon genes-mediated innate immune activation. In the endolysosomal system, ISGylation and ISG15-associated pathways modulate autophagic flux, multivesicular body fate, and exosome secretion in a context-dependent manner. Ribosome-associated co-translational ISGylation links nascent protein surveillance with antiviral defense, whereas lipid droplet-associated ISG15/ISGylation pathways are linked to lipid metabolism and immune signaling. Current evidence supports an organelle-centered view of ISG15 biology but indicates that validated covalent mechanisms remain confined to selected substrates and contexts. Clinical translation will require organelle-resolved ISGylome mapping, substrate-level validation, and standardized biomarker assays before context-selective targeting can be considered.

## 1 Introduction

ISGylation is an interferon (IFN)-induced ubiquitin-like modification process centered on Interferon-Stimulated Gene 15 (ISG15) (1). First identified as the earliest ubiquitin-like protein, ISG15 is covalently attached to lysine residues on target proteins through an enzymatic cascade involving the E1 activating enzyme UBA7, the E2 conjugating enzyme UBE2L6 (UbcH8), and several E3 ligases, such as HERC5 (2, 3). Like ubiquitination, ISGylation is dynamic and reversible, and is reversed primarily by the de-ISGylating enzyme ubiquitin-specific protease 18 (USP18) (4, 5). Research on ISGylation has traditionally centered on its role as a major effector mechanism in host innate immunity, particularly antiviral defense (6–8). Numerous studies have shown that ISGylation can broadly suppress viral infection by directly modifying viral proteins or key host factors, thereby restricting viral entry, replication, assembly, and release (3, 9, 10). However, with continued progress in the field, the functions of ISGylation are now recognized to extend well beyond antiviral activity. Growing evidence indicates that ISGylation, as a widespread post-translational modification (PTM) system, participates extensively in essential cellular processes, including protein translation, autophagy, exosome secretion, DNA damage repair, and immune regulation (1, 11). Proteomic studies have identified more than 1, 100 putative ISGylation substrates, although most modification sites and functional consequences remain unvalidated (3, 12).

**Figure 1:**
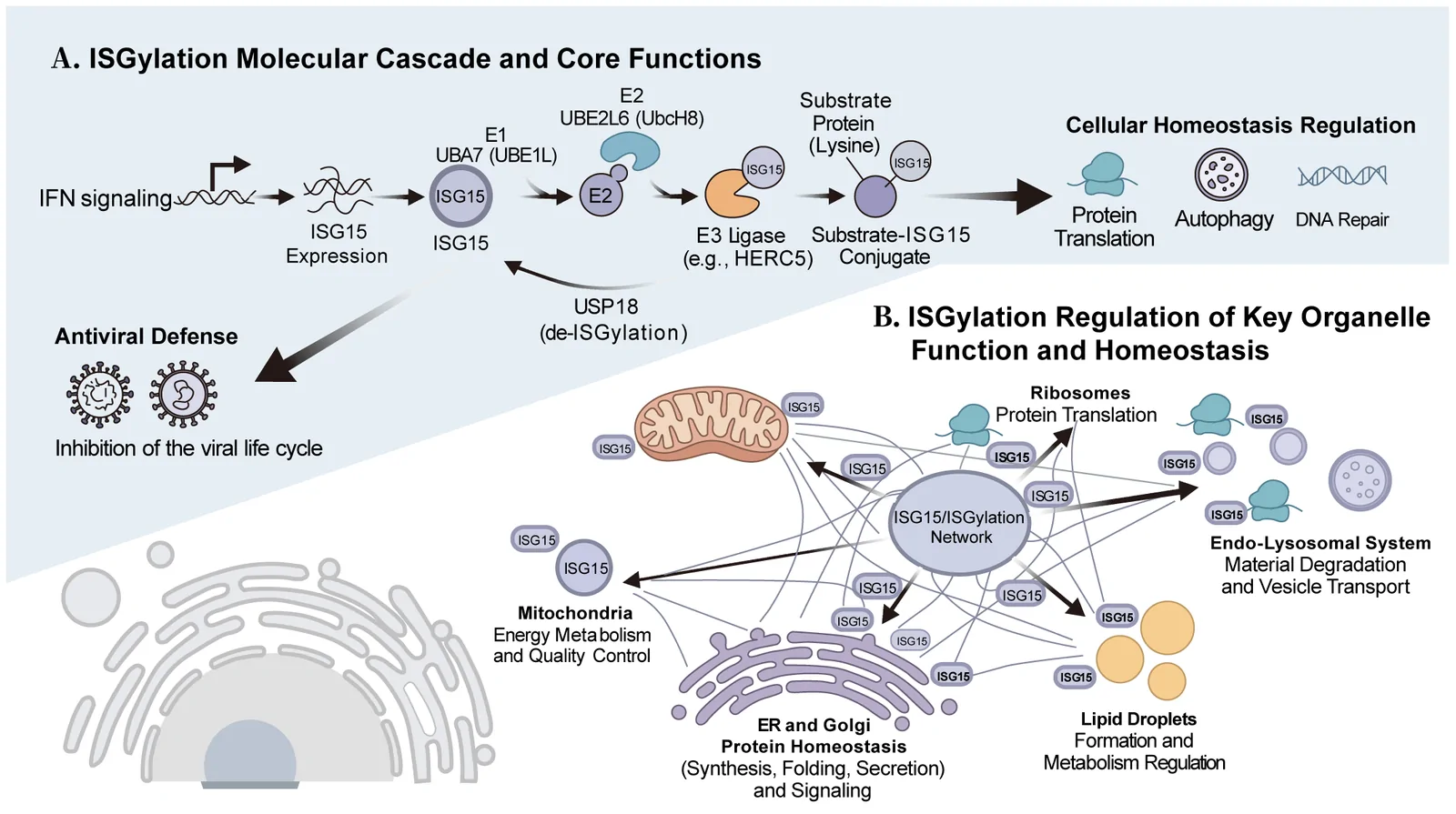
The ISGylation molecular cascade and organelle-centered regulatory framework. **(A)** ISGylation molecular cascade and core functions. IFN signaling induces ISG15 expression and activates the UBA7-UBE2L6-E3 ligase cascade, whereas USP18 and viral de-ISGylases remove ISG15 from modified substrates. This pathway contributes to antiviral defense and the regulation of cellular homeostasis, including protein translation, autophagy, and DNA repair. **(B)** Organelle-centered regulation by ISG15/ISGylation. The schematic illustrates representative mechanisms through which ISG15/ISGylation intersects with mitochondrial quality control, ER-Golgi signaling, vesicular trafficking, and lipid droplet biology. These pathways encompass both direct substrate-level ISGylation and pathway-level ISG15-associated responses, as distinguished in the main text and tables. Arrows indicate activation or trafficking, whereas inhibitory marks represent restriction or degradation. Solid arrows indicate experimentally supported activation, trafficking, or functional outcomes; inhibitory bars indicate inhibition or blockade; dashed arrows indicate indirect, proposed, or correlative relationships.

Organelles are specialized subcellular structures that perform distinct cellular functions, and their proper activity is essential for maintaining cellular homeostasis. In recent years, increasing evidence has suggested that ISG15-related pathways, including covalent ISGylation in selected contexts, participate in the regulation of different organelle functions. From mitochondrial energy metabolism and quality control, to protein homeostasis and signal transduction along the endoplasmic reticulum–Golgi axis, and further to degradation and vesicular trafficking within the endolysosomal system, ISG15-related mechanisms have emerged as context-dependent contributors to organelle function, with covalent ISGylation demonstrated in selected substrate-level studies (6, 13). Notably, mechanistic insights remain dispersed across organelle-specific studies, hindering the development of an integrated understanding of cross-compartmental regulation and PTM crosstalk (**Figure 1**). Because several regulators operate across compartment boundaries, recurring factors are treated here as cross-compartmental nodes rather than as independent mechanisms; detailed mechanisms are introduced once, whereas subsequent sections emphasize only their compartment-specific consequences. This review critically synthesizes recent advances in elucidating how ISGylation and ISG15-associated pathways regulate the function and homeostasis of major organelles, including mitochondria, the endoplasmic reticulum, the Golgi apparatus, the endolysosomal system, ribosomes, and lipid droplets. By structuring the evidence according to subcellular compartments, we seek to clarify how ISG15-related mechanisms contribute to cellular homeostasis and disease pathogenesis. Compared with previous reviews that have primarily emphasized antiviral immunity, global ISG15 biology, or disease-specific associations, this review uses an organelle-centered framework to organize heterogeneous evidence concerning ISG15-related biology. By distinguishing direct substrate-level ISGylation from pathway-level ISG15/IFN-associated responses, we aim to connect organelle-specific mechanisms with disease phenotypes, biomarker development, patient stratification, and therapeutic targeting. This organelle-centered perspective may help identify which ISG15-related events are likely to represent causal disease mechanisms and which are better interpreted as interferon-induced bystander signatures.

### 1.1 Search strategy and evidence classification

For clarity, this review uses the term ISGylation only when covalent conjugation of ISG15 to a substrate is supported or explicitly proposed. We distinguish this from free intracellular ISG15, secreted ISG15, non-covalent ISG15–protein interactions, and USP18-mediated negative regulation of type I interferon signaling, because these mechanisms can produce different, and sometimes opposing, biological outcomes (**Table 1**). To further avoid overinterpreting heterogeneous evidence, we classify ISG15-related findings into three mechanistic evidence levels and separately consider translational hypotheses derived from the available evidence. Level 1 represents direct substrate-level evidence of covalent ISGylation within the experimental system studied, supported by substrate identification and functional evidence; lysine-site mapping and enzyme dependence further strengthen this assignment. Level 2 requires experimental perturbation of ISG15 or a conjugation/deconjugation component together with a linked functional phenotype, but the relevant substrate, modification site, or rescue mechanism remains incomplete. Level 3 refers to associations based primarily on ISG15 expression, interferon signatures, or omics datasets without direct perturbational evidence. Importantly, these levels describe mechanistic strength within the model and species studied and do not imply cross-species conservation or translational readiness. Translational hypotheses are considered separately from the mechanistic evidence levels and refer to proposed clinical applications inferred from existing mechanistic or correlative findings that have not yet been validated in disease-specific models or patient cohorts. This framework enables comparison of mechanistic evidence strength while separately considering potential translational relevance across organelle-associated ISG15 pathways (**Table 2**).

**Table 1:** Conceptual distinctions used to interpret ISG15 biology across organelles.

Term	Operational meaning in this review	Why the distinction matters	Reference(s)
Free intracellular ISG15	Unconjugated ISG15 inside cells	May regulate signaling or protein interactions without proving covalent modification.	(5, 6, 53)
Secreted ISG15	Extracellular ISG15 released from cells	Can have cytokine-like or immunomodulatory effects that differ from intracellular ISGylation.	(6, 7, 11)
Covalent ISGylation	UBA7-UBE2L6-E3-dependent conjugation of ISG15 to lysine residues of substrates	Provides the strongest basis for assigning direct effects to substrate modification.	(1–3, 12)
De-ISGylation	Removal of ISG15 from substrates by USP18 or viral de-ISGylases such as PLpro	Can reverse ISGylation and is therapeutically relevant in viral infection and inflammatory disease.	(4, 35, 36)
USP18 IFN regulation	USP18-mediated suppression of type I interferon signaling independent of catalytic de-ISGylation	Can confound interpretation when ISG15, ISGylation, and interferon tone change simultaneously.	(5, 67)

The categories are operational distinctions used in this review and are not mutually exclusive within a given biological context. References are representative rather than exhaustive.

**Table 2:** Framework for distinguishing mechanistic evidence strength from translational readiness in organelle-associated ISG15 biology.

Evidence category	Definition	Representative examples	Interpretation	Reference(s)
Level 1: Direct substrate-level evidence	Defined covalent substrate ISGylation with functional support in the experimental model	DRP1-mediated mitochondrial fission; MFN1/2 ISGylation in OA-associated mitophagy; HK2/PFK1 ISGylation during CVB3 infection; TSG101-dependent Golgi/MVB regulation; TRIM21 ISGylation; 4EHP ISGylation; zebrafish STING ISGylation; UBE2L6–VCP–ATGL axis	Strongest mechanistic evidence within the experimental model, but does not imply cross-species conservation or near-term clinical actionability	(19, 23, 25, 51, 54, 56, 62, 72, 74)
Level 2: Pathway-level evidence	Experimental perturbation of ISG15 or a conjugation/deconjugation component is linked to a functional phenotype, but the relevant substrate, modification site, or rescue mechanism remains incomplete	PaCSC mitochondrial ISGylation/mitophagy; ISG15–NOX4-associated oxidative stress; HERC5/HERC6-associated co-translational ISGylation with unresolved substrate-specific consequences; non-covalent ISG15–HDAC6 regulation; selected RNF213- and ACSL4-related lipid-droplet pathways; selected ERAD/UPR-associated ISG15 pathways	Supports mechanistic and disease modeling, but requires substrate/site-specific or mechanism-appropriate validation before therapeutic interpretation	(3, 12, 18, 28, 35–42, 55, 75, 76)
Level 3: Correlative evidence	Association based primarily on ISG15 expression, interferon signatures, or omics datasets without direct ISG15-pathway perturbation	COVID-19 and coronary microembolization-associated mitochondrial signatures; disease-associated ribosome/translation signatures; leishmaniasis-associated ISG15 signatures; ovarian cancer-associated exosome phenotype without substrate-specific validation; ankylosing-spondylitis-associated lipid signatures	Useful for hypothesis generation and biomarker discovery, but causality and organelle-specific covalent ISGylation should not be assumed	(26, 27, 43, 49, 50, 63, 70, 71, 77)
Translational hypothesis	Clinical application is assessed separately from mechanistic evidence level	Near-term biomarker/pharmacodynamic validation; medium-term disease- or substrate-selective interventions; exploratory global ISG15/USP18-pathway manipulation	Translational readiness depends on assay feasibility, disease specificity, human validation, target selectivity, therapeutic window, and safety rather than evidence level alone	(18, 19, 28, 51, 55, 62, 74, 78, 79)

Evidence levels describe mechanistic strength within the experimental model and species studied and do not imply cross-species conservation or translational readiness. Level 1 denotes defined covalent substrate ISGylation with functional support; Level 2 denotes perturbation-supported pathway evidence with incomplete substrate/site or rescue validation; and Level 3 denotes association without direct ISG15-pathway perturbation. Translational readiness is assessed separately from mechanistic evidence strength. References are representative rather than exhaustive.

The literature was surveyed in PubMed, Web of Science, and Scopus through August 2026 using combinations of the terms ISG15, ISGylation, USP18, UBA7, UBE2L6, HERC5, HERC6, organelle, mitochondria, endoplasmic reticulum, Golgi, autophagy, lysosome, exosome, ribosome, lipid droplet, biomarker, and therapy. The narrative selection prioritized English-language, peer-reviewed articles, particularly those providing evidence of covalent substrate ISGylation, organelle localization, disease relevance, biomarker potential, or therapeutic implications (**Supplementary Tables 1, 2**). As this is a narrative rather than a systematic review, no formal meta-analysis or risk-of-bias assessment was conducted. Therefore, the evidence categories used here should be interpreted as a qualitative framework for comparing mechanistic strength rather than as a formal grading system. This evidence framework guided the interpretation of organelle-specific ISGylation mechanisms throughout the review.

Using this framework, the following sections examine ISGylation across major organelle systems, beginning with mitochondrial dynamics, metabolism, and quality control.

## 2 ISGylation and mitochondria: a hub for dynamics, metabolism, and quality control

### 2.1 Regulation of mitophagy and organelle quality control

Mitophagy is a selective autophagic process that removes damaged or superfluous mitochondria and is essential for maintaining mitochondrial network integrity and cellular homeostasis (14). In certain disease models, the ISGylation system has emerged as an important regulator of mitophagy and mitochondrial quality control, particularly when interferon signaling, proteostasis, and mitochondrial stress are disrupted. In ataxia-telangiectasia (A-T), aberrantly elevated ISG15 signaling has been reported as a candidate mechanism contributing to defective mitophagy in A-T cell models (15, 16). Studies have shown that constitutively high ISG15 expression in A-T cells inhibits ubiquitin-dependent protein degradation pathways, thereby disrupting the efficient clearance of damaged mitochondria (15, 17). More specifically, ISG15 can antagonize SUMO2 modification of the mitochondrial outer membrane fusion proteins mitofusins (Mfns), impairing the congregation of damaged mitochondria in the perinuclear region and preventing the formation of functional pre-autophagosomal structures, or mito-aggresomes, thereby interrupting the initial step of mitophagy (16). Knocking down ISG15 restores the capacity of damaged mitochondria to congregate in A-T cells, reduces oxidative stress, and increases the level of healthy mitochondria (14–16). In A-T cell models, excessive ISG15 signaling is associated with impaired mitophagy and mitochondrial dysfunction; its causal relevance and therapeutic tractability in other neurodegenerative disorders remain to be established.

Beyond neurodegeneration, ISGylation-dependent control of mitophagy also contributes to metabolic adaptability in tumor settings. The marked plasticity and drug resistance of pancreatic cancer stem cells (PaCSCs) are closely linked to their efficient mitochondrial quality-control system. Research indicates that ISG15 expression and protein ISGylation levels are significantly upregulated in PaCSCs, a feature that is essential for maintaining their metabolic plasticity (18). CRISPR-mediated knockout of *ISG15* reduces mitochondrial ISGylation in PaCSCs, leading to the accumulation of dysfunctional mitochondria, defective mitophagy, and a subsequent decline in oxidative phosphorylation (OXPHOS) capacity. This metabolic impairment sensitizes PaCSCs to drugs targeting mitochondrial metabolism, such as metformin (18). ISG15-dependent mitochondrial quality control supports PaCSC metabolic plasticity and sensitivity to mitochondrial metabolic stress, although the relevant ISGylated substrates remain incompletely defined.

In inflammatory and degenerative disease settings, ISGylation similarly intersects with canonical PINK1/Parkin quality-control pathways through PTM crosstalk. In chondrocytes derived from osteoarthritis (OA) patients, inflammatory cytokine-induced IRF1 promotes *PARP12* transcription. PARP12 interacts with ISG15 and enhances the ISGylation of MFN1/2. This modification inhibits MFN ubiquitination and SUMOylation, thereby disrupting PINK1/Parkin-mediated mitophagy and aggravating cartilage degeneration (19). The PARP12–ISG15–MFN1/2 axis provides a mechanistic link between inflammatory signaling and defective mitophagy in OA chondrocytes, but requires validation in independent OA models.

Pathogens can further remodel mitochondrial quality control by hijacking, antagonizing, or redirecting ISGylation-linked checkpoints. Viral infections often regulate mitophagy by hijacking or interfering with ISGylation. Pathogens can remodel mitochondrial innate immune signaling and quality-control pathways through mechanistically distinct routes. In ASFV infection, MGF_360-4L interacts with MDA5 and recruits the selective autophagy receptor SQSTM1/p62 to promote MDA5 degradation, thereby impairing MDA5–MAVS signaling and altering mitochondria-associated antiviral responses (20). Separately, SARS-CoV-2 PLpro antagonizes ISG15-dependent activation of MDA5 through its de-ISGylating activity, providing a distinct mechanism by which viral proteins interfere with the ISG15–MDA5 axis (21). These findings illustrate that viruses can suppress MDA5 signaling either through selective autophagic degradation or through direct antagonism of ISGylation, but these mechanisms should not be considered equivalent. MGF_360-4L therefore links MDA5 degradation and impaired MDA5 ISGylation to ASFV evasion of mitochondrial innate immune signaling.

Consistently, host cytokine programs can counterbalance this axis by adjusting ISG15 abundance to restore autophagic competence. In an ulcerative colitis model, the anti-inflammatory cytokine IL-10 enhanced autophagy by downregulating ISG15 expression, thereby reducing intestinal epithelial cell apoptosis and oxidative stress while repairing the intestinal mucosal barrier (22). This effect could be reversed by ISG15 overexpression, indicating that ISG15 is a key downstream target of the protective effect of IL-10 and plays a significant inhibitory role in autophagy regulation. Across these disease models, altered ISG15 abundance intersects with autophagy and mitochondrial stress, but the direction and underlying mechanism vary according to biological context.

### 2.2 Balancing mitochondrial dynamics: fine-tuning fission and fusion

Because mitochondrial morphology and turnover are tightly interconnected, dynamic fission and fusion programs often influence, and respond to, mitophagy and broader quality-control decisions. Mitochondria form a highly dynamic network whose architecture is continuously maintained and remodeled through coordinated fission and fusion. The balance between these processes is essential for mitochondrial function, quality control, and cellular adaptation to changing physiological demands. As a key post-translational modification, ISGylation can modify or stabilize selected proteins that control mitochondrial dynamics, thereby influencing mitochondrial morphology and function in specific contexts. DRP1 has been identified as a direct substrate of ISG15 modification (23). HERC5-mediated ISGylation of DRP1 significantly promotes mitochondrial fission. Conversely, de-ISGylation of DRP1, such as through overexpression of the SARS-CoV-2 de-ISGylating enzyme PLpro, leads to excessive mitochondrial fusion with the formation of filamentous networks, accompanied by reduced total DRP1 protein and efflux of mitochondrial DNA (mtDNA) (23).

ISGylation interacts in complex ways with other PTMs, together shaping DRP1 function. For example, phosphorylation of DRP1 at Ser616 is required for its recruitment to mitochondria to execute fission, whereas subsequent ISGylation confers functional activity on phosphorylated DRP1 at fission sites (23). De-ISGylated DRP1 is more susceptible to TRIM25-mediated ubiquitination, but not ubiquitination by Parkin or MITOL, followed by proteasomal degradation (23). This finding supports a role for ISGylation in maintaining DRP1 protein stability and functional activity. Dysregulated DRP1 ISGylation may therefore contribute to altered mitochondrial dynamics, although its disease-specific relevance in neurodegenerative disorders requires further validation.

MFN1/2 also link mitochondrial quality control to organelle dynamics. As described in Section 2.1, PARP12-associated MFN1/2 ISGylation in OA chondrocytes alters competing ubiquitin and SUMO modifications and impairs PINK1/Parkin-mediated mitophagy (19); however, that study did not directly test mitochondrial fusion. Furthermore, in A-T cells, elevated ISG15 has been linked to altered ER–mitochondria contacts and disturbed calcium homeostasis, potentially through interference with ubiquitin-dependent regulation of MFN2 and other ER–mitochondria contact components. These changes may contribute to mitochondrial dysfunction and calcium-related stress in A-T (15, 24). Direct evidence establishes DRP1-dependent mitochondrial fission and MFN1/2-associated mitophagy; whether MFN1/2 ISGylation directly alters mitochondrial fusion remains unknown. A potential relationship among ISG15-dependent MFN2 regulation, ER–mitochondria contacts, and calcium homeostasis remains mechanistically unresolved.

### 2.3 Remodeling mitochondrial metabolism, oxidative stress, and energy production

The functional consequences of altered mitochondrial quality control and dynamics ultimately manifest in metabolic outputs, including ATP production, redox balance, and stress resilience, thereby linking ISG15-related pathways to organismal phenotypes and disease severity. The role of ISG15/ISGylation-related pathways in mitochondrial regulation appears to extend beyond dynamics and quality control. It is also closely involved in core metabolic processes, including oxidative phosphorylation (OXPHOS), glycolysis, fatty acid oxidation, and the production and clearance of reactive oxygen species (ROS), placing it at the intersection of energy production and oxidative stress homeostasis. Under stress conditions such as viral infection, ISG15-mediated metabolic reprogramming is critical for host defense. A study of coxsackievirus B3 infection in the heart showed that ISGylation directly modifies two key rate-limiting glycolytic enzymes, Hexokinase 2 (HK2) and Phosphofructokinase muscle type (PFK1), thereby inhibiting their activity (25). By suppressing glycolysis, ISGylation allows cardiomyocytes experiencing nutrient scarcity during infection to reduce their reliance on glucose and instead increase mitochondrial OXPHOS capacity, ensuring an adequate ATP supply to counteract virus-induced cardiac atrophy and dysfunction (25). In coxsackievirus B3-infected cardiomyocytes, ISGylation of HK2 and PFK1 suppresses glycolysis and supports compensatory OXPHOS, defining a stress-adaptive metabolic mechanism.

Disease-associated alterations in the ISG15 pathway show model-dependent and potentially bidirectional associations with mitochondrial respiratory function and oxidative stress. In patients with severe COVID-19, markedly elevated ISG15 levels in peripheral blood mononuclear cells are inversely correlated with severely impaired mitochondrial respiration, which in turn positively correlates with patient mortality and the severity of long COVID symptoms (26). By contrast, in a rat model of coronary microembolization, cardiac tissue exhibited mitochondrial energy-metabolism dysfunction, characterized by substantially reduced ATP levels and mitochondrial membrane potential, together with markedly increased ROS production. Transcriptomic analysis revealed the downregulation of multiple mitochondria-related genes, including *ISG15*, accompanied by pronounced suppression of pathways associated with OXPHOS and the electron transport chain (27). In an acute kidney injury (AKI) model, the dopamine D4 receptor (DRD4) downregulated its target gene *ISG15*, reduced ISGylation of NADPH oxidase 4 (NOX4), and promoted NOX4 ubiquitination and degradation. This process decreased ROS production, enhanced mitochondrial bioenergetics, and exerted a renoprotective effect (28). Across the examined models, ISG15 expression or ISGylation is associated with mitochondrial respiration and oxidative stress in different directions. However, the COVID-19 and coronary microembolization findings are primarily correlative or transcriptomic, and therefore do not establish whether altered ISG15 expression is a cause or a consequence of mitochondrial dysfunction.

**Figure 2:**
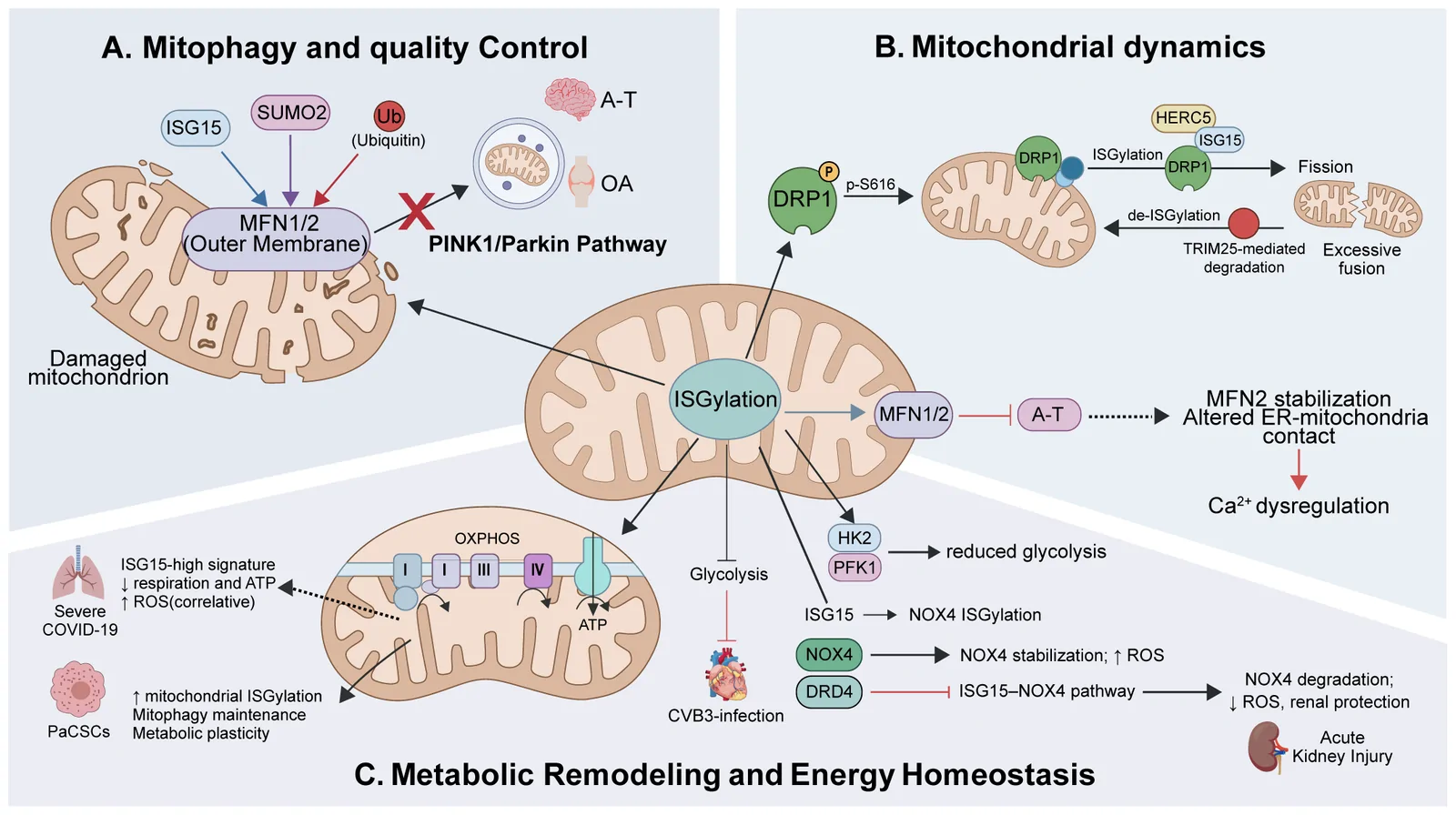
ISGylation integrates mitochondrial quality control, fission-fusion dynamics, and metabolic rewiring. **(A)** Mitophagy and mitochondrial quality control, including MFN1/2-associated regulation and the PINK1/Parkin pathway. **(B)** Mitochondrial dynamics, highlighting DRP1-mediated mitochondrial fission, MFN1/2-associated regulation, and ER-mitochondria contacts. Direct effects of MFN1/2 ISGylation on mitochondrial fusion have not been established. **(C)** Metabolic remodeling and energy homeostasis associated with mitochondrial ISGylation, including changes in oxidative phosphorylation, glycolysis, and redox regulation. Direct substrate-level mechanisms include DRP1- and MFN-related regulation, whereas COVID-19-associated mitochondrial respiratory impairment and selected metabolic disease signatures should be interpreted primarily as correlative or pathway-level evidence unless substrate-specific ISGylation is demonstrated. Red inhibitory symbols indicate blocked degradation, trafficking, or pathway activity; arrows indicate activation, stabilization, or metabolic flux. Solid arrows indicate experimentally supported activation, trafficking, or functional outcomes; inhibitory bars indicate inhibition or blockade; dashed arrows indicate indirect, proposed, or correlative relationships.

Loss of ISG15 also gives rise to complex mitochondrial metabolic phenotypes. ISG15-deficient human macrophages and endothelial cells display reduced oxidative phosphorylation and β-oxidation capacity, decreased expression of mitochondrial biogenesis-related genes involving complexes II–V, and elevated oxidative stress (29). In macrophages, ISG15 deficiency compromises the regulation of lipid metabolism during vaccinia virus (VACV) infection, as indicated by fewer lipid droplets and increased lipolysis (30). It also disrupts macrophage polarization and NO production, thereby weakening viral clearance capacity through a process closely associated with impaired mitochondrial function (31). ISG15/ISGylation-related pathways further shape mitochondrial metabolism across diverse disease contexts. For example, in polycystic ovary syndrome, IGF2BP2 upregulates ISG15 through m6A modification, promoting mitochondrial respiration and glycolysis in granulosa cells (32). In cervical cancer, ISG15 has been associated with apoptosis involving mitochondrial and ER-stress pathways (33). Current evidence includes direct ISGylation of selected metabolic enzymes and indirect or correlative associations between the ISG15 axis, mitochondrial respiration, and redox homeostasis. Its context-specific regulation may affect cellular adaptation to physiological and pathological stress (**Figure 2**). These mitochondrial studies identify several testable molecular and functional readouts for future preclinical validation. Mitochondrial ISGylation may have clinical relevance in disorders in which defective mitophagy, redox imbalance, or metabolic plasticity drives disease progression. Relevant readouts for future validation include DRP1 and MFN ISGylation, PINK1/Parkin pathway activity, mitochondrial respiration, ROS burden, and sensitivity to metabolic stressors such as metformin. Substrate-specific interventions that normalize DRP1 or MFN modification could, in theory, restore mitochondrial dynamics, whereas broader suppression of ISG15 may impair antiviral defense. Patient selection, disease stage, and the distinction between protective and pathogenic ISGylation will therefore be essential before this axis can be therapeutically developed. Overall, mitochondrial ISG15 biology is supported by evidence of markedly different mechanistic strength and direction. The most firmly established covalent mechanisms include HERC5-dependent ISGylation of DRP1, MFN1/2 ISGylation in osteoarthritis chondrocytes, and ISGylation of HK2 and PFK1 during coxsackievirus B3 infection. By contrast, a direct effect of MFN1/2 ISGylation on mitochondrial fusion, the identities of the mitochondrial substrates required for PaCSC mitophagy, and the proposed link among ISG15, MFN2, ER–mitochondria contacts, and calcium homeostasis remain incompletely resolved. Apparent discrepancies—such as ISG15 supporting mitophagy and metabolic fitness in PaCSCs but impairing mitophagy in A-T and OA models, or both increased and decreased ISG15 states being associated with mitochondrial respiratory dysfunction—likely reflect differences in cell type, disease state, interferon tone, and whether free ISG15, global ISGylation, or a defined substrate modification is being measured. Decisive experiments should combine conjugation-deficient ISG15 rescue, substrate-specific lysine mutants, and matched measurements of mitophagic flux, mitochondrial morphology, respiration, and redox state in disease-relevant models.

## 3 ISGylation and the ER–Golgi axis: convergence of proteostasis and immune signaling

### 3.1 Regulation of ER stress, protein processing, and degradation

The endoplasmic reticulum (ER) is the principal site of protein synthesis, folding, and modification, and ER homeostasis is essential for normal cellular function. Accumulation of unfolded or misfolded proteins within the ER lumen triggers ER stress and activates the unfolded protein response (UPR) (34). The ISG15/ISGylation system is closely connected to the regulation of ER homeostasis and appears to play a dual role, particularly in pathological settings such as viral infection and tumorigenesis. On one hand, certain viral proteins can exploit or alter ISGylation of ER-associated proteins or perturb ER-associated ISG15 pathways for their own benefit. For instance, the SARS-CoV-2 non-structural protein 3 (Nsp3) contains a papain-like protease (PLpro) with both deubiquitinating and de-ISGylating activities (35, 36). When anchored to the ER membrane, PLpro can act as a deubiquitinating enzyme that stabilizes ERAD substrates such as INSIG-1 and can also affect the processing of ER-resident lipid-regulatory proteins SREBP-1 and SREBP-2, thereby remodeling lipid-metabolic pathways (35). These activities indicate that viral PLpro can reshape the cellular metabolic environment by perturbing ER-associated ubiquitin- and ISG15-linked proteostasis and lipid-regulatory pathways (35, 36). Furthermore, several SARS-CoV-2 structural proteins, including S, M, and E, can induce ER stress and upregulate ER stress-related genes, including interferon-stimulated genes such as ISG15 (34, 37).

ISG15-associated interferon programs and coronavirus Nsp3–host interactions also converge on ER stress and UPR signaling. In nasopharyngeal carcinoma, RIG-I expression correlates with sensitivity to chemoradiotherapy. RIG-I overexpression can activate the ER stress response pathway, involving key molecules such as ATF6 and XBP1, thereby promoting apoptosis (38). ISG15 expression increases as part of the IFN/JAK2-associated response, but this finding should be interpreted as a pathway-level association rather than direct evidence that covalent ISGylation regulates ER stress. In addition, comparative analysis of the coronavirus Nsp3 interactome showed that the N-terminal fragment of SARS-CoV-2 Nsp3 can directly bind ATF6, a key UPR regulator, and inhibit the stress response mediated by ATF6 (39, 40). IFN/ISG15-associated signaling and Nsp3–ATF6 interactions occur within a shared ER-stress context; however, direct evidence for ER-localized covalent ISGylation remains limited.

Viral proteins can also modulate host ISGylation by targeting ER-associated ISGylation enzymes and proteostasis factors. Human cytomegalovirus pUL50 is an ER-localized transmembrane protein that recruits the ER-associated E3 ubiquitin ligase RNF170 to promote ubiquitination and proteasomal degradation of the ISGylation-activating enzyme UBA7, thereby suppressing global cellular ISGylation levels (41). Interestingly, the function of pUL50 itself is regulated by a short isoform, UL50-p26, translated from an internal start codon; this isoform inhibits pUL50-induced degradation of VCP/p97, a key unfoldase in the ERAD pathway (42). This intricate regulatory network shows that viruses can precisely manipulate ER protein processing and degradation systems through multiple mechanisms, with ISGylation acting as a regulatory node. Collectively, current evidence links ER proteostasis to the ISG15 system through viral regulation of ERAD components and ISGylation enzymes, UPR-associated interferon responses, and viral immune antagonism (35, 38–41, 43). However, direct substrate-level evidence for ER-localized covalent ISGylation remains limited.

### 3.2 Mediating immune signaling molecules localized to the ER–Golgi axis

In addition to its role in proteostasis control, the ER–Golgi axis serves as a spatial platform where innate immune signaling components assemble, traffic, and become activated; consequently, membrane localization and vesicular transport are integral determinants of signal amplitude and duration. Stimulator of Interferon Genes (STING) is a central adaptor protein that resides in the ER. Upon activation by the second messenger cyclic GMP-AMP, STING translocates from the ER to the Golgi apparatus, where it recruits and activates TBK1 and ultimately initiates type I interferon (IFN-I) production (44).

Several studies define the ER–Golgi spatial regulation of STING signaling without directly demonstrating covalent ISGylation of STING or its trafficking regulators. Several studies establish the importance of ER–Golgi localization for STING signaling: ASFV p17 disrupts STING–TBK1/IKKϵ signaling, whereas ER-localized STING and RACK1-dependent perinuclear STING aggregation can enhance antiviral type I IFN responses in distinct experimental systems (45–47). However, none of these studies demonstrates covalent ISGylation of STING, RACK1, or ASFV p17, and they should therefore be interpreted as evidence for ER–Golgi spatial regulation rather than direct ISGylation mechanisms.

Beyond STING, other immune-related molecules associated with ER–Golgi or organelle-based signaling may intersect with ISGylation-related regulation, although the evidence varies from direct substrate modification to pathway-level association. NLRP3 is regulated by multiple post-translational modifications, including HERC-mediated ISGylation, whereas a separate study showed that NLRP3 activation involves recruitment to phosphatidylinositol-4-phosphate-enriched dispersed trans-Golgi network platforms. Taken separately, the available studies support HERC-mediated NLRP3 ISGylation and trans-Golgi-network-dependent NLRP3 activation, but not Golgi-localized NLRP3 ISGylation (13, 48). Disease-associated IFN/ISG15 signatures have also been reported in leishmaniasis (49, 50), but these findings remain Level 3 associations without identified ISGylated substrates or ER–Golgi-specific mechanisms. Evidence across the ER–Golgi axis spans direct substrate ISGylation, pathway-level effects on STING localization and trafficking, and correlative IFN/ISG15 responses.

### 3.3 Impact on Golgi-associated protein trafficking and viral assembly

Because effective immune signaling along the ER–Golgi axis depends on tightly regulated trafficking, ISGylation-associated “traffic control” can shape host signaling outcomes while also constraining viral maturation routes that rely on the secretory pathway. As a central regulatory platform for protein sorting, modification, and trafficking, the Golgi apparatus is essential to both the cellular secretory pathway and the viral life cycle. By modulating Golgi function, the ISGylation system places substantial constraints on viral protein maturation and virion assembly and release. A study of influenza A virus (IAV) identified a Golgi-localized checkpoint comprising ISG15 and TSG101, a core component of the ESCRT-I complex, that specifically restricts viral glycoprotein trafficking (51). In cells treated with type I interferon, activation of the ISGylation pathway leads to ISGylation of TSG101. This modification disrupts the efficient transport of IAV hemagglutinin (HA) from the Golgi apparatus to the plasma membrane, thereby limiting the release of infectious viral particles. Cells in which TSG101 was knocked out using CRISPR-Cas9 technology likewise showed defective HA surface display and reduced virus release, further confirming the importance of TSG101 in this process (51). This study proposed a mechanism by which ISGylation-mediated Golgi trafficking checkpoints restrict viral maturation pathways.

Beyond directly affecting viral protein trafficking, ISG15-associated responses may also influence cellular functions indirectly by regulating the expression and localization of Golgi-associated proteins. The Golgi-localized N-α-acetyltransferase NAA60 suppresses IFNα-dependent ISG expression, including ISG15, during IAV infection (52). This finding supports pathway-level regulation of the ISG15 response but does not demonstrate Golgi-localized covalent ISGylation.

**Figure 3:**
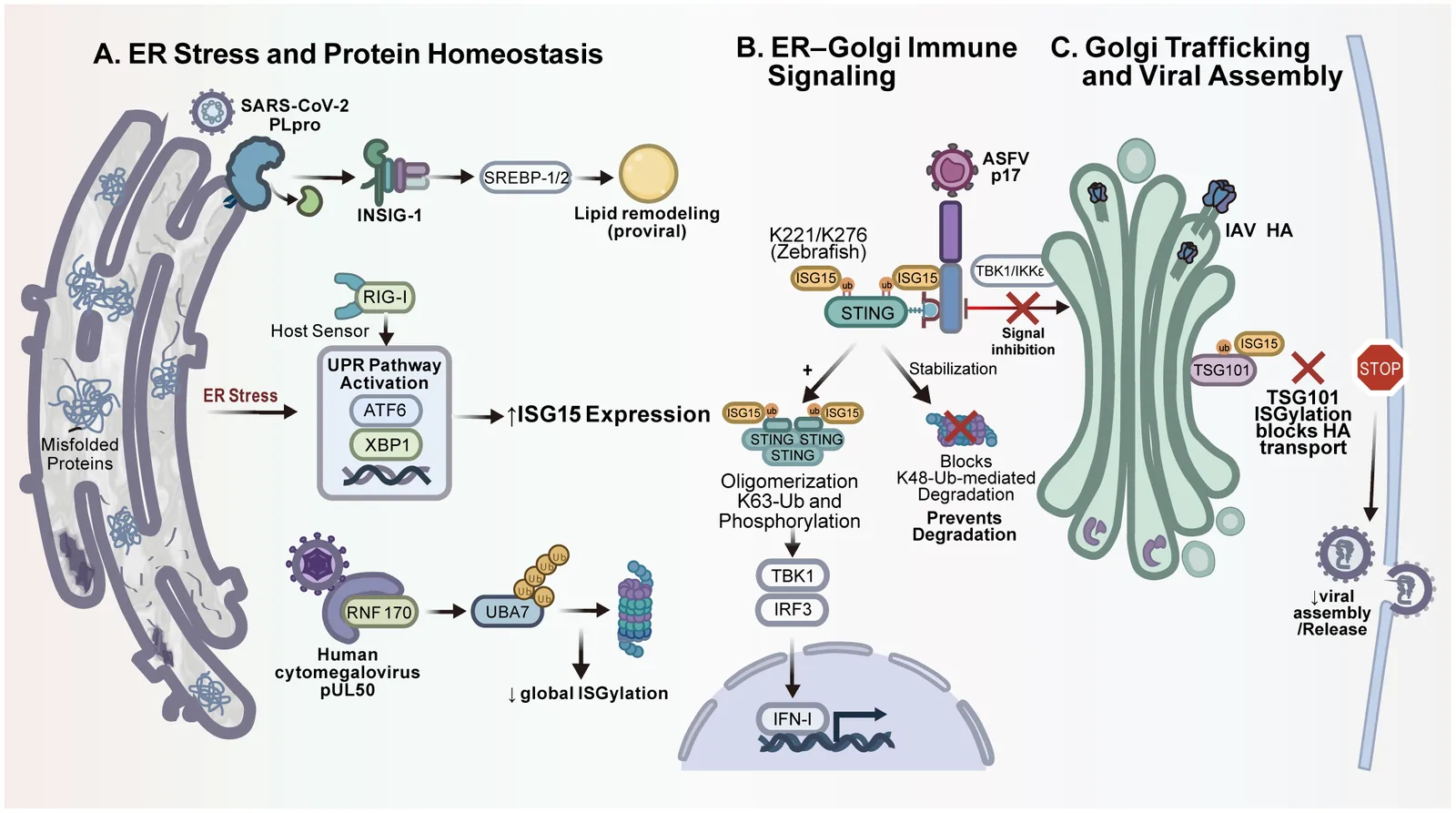
ISGylation regulates ER–Golgi proteostasis, immune signaling, and trafficking. **(A)** ER stress and protein homeostasis. Viral factors such as SARS-CoV-2 PLpro and HCMV pUL50 can remodel ER-associated ubiquitin/ISG15-linked proteostasis and suppress host ISGylation enzymes. **(B)** ER-Golgi immune signaling. STING trafficking from the ER to the Golgi provides a spatial platform for TBK1/IRF3 activation and type I interferon induction, whereas viral or host regulatory factors can alter STING stability, ubiquitination, localization, and signaling competence. **(C)** Golgi trafficking and viral assembly. At the Golgi, ISGylation of TSG101 can restrict influenza A virus hemagglutinin trafficking and limit viral assembly and release. Direct substrate-level TSG101 ISGylation and zebrafish STING ISGylation should be distinguished from broader pathway-level evidence concerning mammalian STING trafficking and interferon-associated responses. Solid arrows indicate experimentally supported activation, trafficking, or functional outcomes; inhibitory bars indicate inhibition or blockade; dashed arrows indicate indirect, proposed, or correlative relationships.

During PRV infection, free ISG15 enhances type I IFN signaling and restricts viral replication, representing a free-ISG15 signaling effect rather than Golgi-localized covalent ISGylation (53). In zebrafish, ISGylation of STING at Lys221 and Lys276 promotes STING oligomerization, K63-linked ubiquitination and phosphorylation, and appropriate Golgi localization, thereby supporting downstream immune signaling (54). Because these modification sites were identified in zebrafish STING, their conservation and functional relevance in human STING remain to be established. This finding therefore constitutes Level 1 substrate evidence within the zebrafish model, but not Level 1 evidence for human STING; its relevance to human STING remains exploratory. Some viral proteins, such as ASFV p17, are themselves localized to the Golgi and interfere with immune signaling by interacting with host proteins such as STING, a process that may also be related to disruption of normal Golgi trafficking functions (45). At the Golgi, direct evidence is strongest for TSG101 ISGylation during IAV infection, whereas effects on immune-adaptor localization remain context- and species-dependent (45, 51, 52, 54) (**Figure 3**). This ER–Golgi-centered view has practical implications for biomarker development and therapeutic targeting. The ER–Golgi axis links ISG15/ISGylation-related pathways to innate immune signaling, viral protein maturation, and proteostasis. Readouts that could be evaluated include STING stability and localization, TBK1/IRF3 activation, ER-stress markers, global ISGylation, and the ISG15–TSG101 trafficking checkpoint. In contrast, direct manipulation of host STING-associated pathways will require careful consideration of antiviral benefits relative to inflammatory toxicity, particularly in patients with elevated interferon tone. Evidence for covalent ISGylation along the ER–Golgi axis remains uneven. The strongest substrate-level evidence currently concerns the TSG101-dependent Golgi trafficking checkpoint, whereas much of the ER literature demonstrates pathway-level interactions between viral proteins, ER proteostasis, and the ISG15 machinery rather than direct ISGylation of ER-resident substrates. Similarly, mammalian studies establish the importance of STING localization and ER-to-Golgi trafficking but do not demonstrate covalent ISGylation of STING in those systems; the reported ISGylation sites in zebrafish STING therefore require validation for conservation and function in human cells. A major unresolved question is whether ISGylation directly remodels UPR or ERAD components or instead influences these pathways indirectly through interferon signaling or viral de-ISGylases. Organelle-resolved ISGylome profiling under defined ER-stress conditions, human STING lysine-mutant rescue experiments, and separation-of-function PLpro mutants that discriminate deubiquitinating from de-ISGylating activity would help resolve these possibilities.

## 4 ISGylation and the endolysosomal system: regulation of autophagic flux, cargo degradation, and exosome secretion

### 4.1 Regulation of autophagic flux and lysosomal function

The autophagy–lysosomal pathway is the principal intracellular system for cargo degradation and recycling and is essential for removing damaged organelles, misfolded proteins, and invading pathogens. ISG15-related mechanisms, including non-covalent ISG15 interactions and covalent ISGylation in selected contexts, exert complex regulatory effects at several stages of this pathway, particularly on autophagic flux, which encompasses autophagosome formation, fusion with lysosomes, and final cargo degradation. In the pathological context of Alzheimer’s disease, ISG15 expression is markedly elevated in both patient brain tissue and disease models (55). Overexpressed ISG15 binds to histone deacetylase 6 (HDAC6) through its C-terminal LRLRGG motif, thereby inhibiting HDAC6 activity. This increases cortactin acetylation, disrupts the recruitment of cortactin and F-actin to lysosomes, and ultimately impairs autophagosome–lysosome fusion. The resulting blockade of autophagic flux promotes tau protein accumulation and neuronal damage. Conversely, ISG15 knockdown restores HDAC6 activity, enhances autophagic flux, and improves cognitive function (55). This mechanism reveals a “vicious cycle” in which ISG15 aggravates Alzheimer’s disease pathology by suppressing autophagic flux.

The effects of ISG15-related mechanisms on autophagic flux are not uniformly inhibitory but instead depend on whether the relevant mechanism involves free ISG15, non-covalent protein interactions, or covalent substrate ISGylation. During IFN-β-induced autophagy, the E3 ligase HERC5 catalyzes ISGylation of TRIM21 at residues K260 and K279, enhancing the intrinsic E3 ligase activity of TRIM21 and promoting K63-linked ubiquitination of the autophagy adaptor p62 (56). This modification prevents p62 oligomerization and its targeting to autophagosomes, thereby regulating the autophagic process. In contrast, another study found that ISG15 and its conjugates can interact with HDAC6 and p62, recruiting these proteins to insoluble cytosolic fractions, particularly after proteasome inhibitor treatment (57). This suggests that, under stress conditions, ISG15 conjugates may tag proteins for routing toward autophagic degradation through HDAC6 and p62. Furthermore, after ischemic stroke, upregulated NLRC5 protein in microglia binds ISG15 through its CARD domain and protects it from autophagic–lysosomal degradation. Stabilized ISG15 disrupts lysosomal function and blocks autophagic flux, thereby promoting neuroinflammation (58).

Viruses have also evolved strategies to exploit or counteract the regulation of autophagy by ISGylation. The nonstructural protein Nsp11 of porcine reproductive and respiratory syndrome virus degrades *ISG15* mRNA through its endoribonuclease activity, partly in a manner dependent on the autophagy–lysosomal system, to antagonize the host antiviral response (59). The same multifunctional SARS-CoV-2 PLpro discussed in Section 3.1 also remodels autophagy, stabilizing the N-degron substrate Arg-HSPA5 and disrupting reticulophagy through ULK1 cleavage and reduced LC3 lipidation (35, 60, 61). ISG15 influences autophagic flux through mechanistically distinct routes, including covalent substrate modification, non-covalent protein interactions, and viral remodeling of the autophagy machinery.

### 4.2 Context-dependent regulation of MVB–lysosome fate and exosome secretion

Because lysosomes also serve as the terminal destination for endosomal cargo, ISGylation-driven changes in lysosomal fusion can redistribute flux between intracellular degradation and extracellular vesicle release. Multivesicular bodies (MVBs) are late endosomes that contain numerous intraluminal vesicles (ILVs). MVBs have two principal fates: they either fuse with lysosomes, resulting in degradation of their contents, or fuse with the plasma membrane, releasing their ILVs as “exosomes” that mediate intercellular communication (62). ISGylation has been identified as an important regulator of MVB fate and exosome secretion in IFN-activated and other selected cellular contexts. Available evidence supports the view that activation of ISGylation markedly inhibits exosome secretion (62). Upon stimulation, such as with IFN, increased intracellular ISGylation reduces MVB numbers and impairs exosome release. This inhibitory effect depends on the covalent conjugation activity of ISG15. Consistently, exosome secretion is also suppressed in mouse models expressing catalytically inactive USP18 (62).

TSG101, already discussed in Section 3.3 as a Golgi trafficking substrate during IAV infection, also participates in a mechanistically distinct endosomal pathway controlling MVB fate. In IFN-activated settings, TSG101 ISGylation promotes MVB-associated protein aggregation and lysosomal degradation and is sufficient to suppress exosome secretion (62). However, this regulatory relationship may be rewired in a context-dependent manner in certain cancer backgrounds. Such regulation has been observed across diverse physiological and pathological settings. In ovarian cancer, for example, elevated ISG15 expression increases exosome secretion by reducing endosome–lysosome fusion, thereby promoting cancer cell migration and invasion. This finding indicates that elevated ISG15 expression can promote exosome secretion in ovarian cancer, but it does not establish that covalent ISGylation of TSG101 or other MVB-associated proteins mediates this effect (63). These apparently opposing observations indicate that ISG15-associated regulation of exosome biogenesis is context-dependent; however, they should not be interpreted as opposite effects of the same covalent ISGylation mechanism because the ovarian cancer study primarily assessed ISG15 expression rather than substrate-specific ISGylation.

Several factors may explain these divergent outcomes. First, immune, stromal, and tumor cells differ in their MVB-sorting machinery, lysosomal capacity, and dependence on ESCRT components. Second, elevated ISG15 expression does not necessarily reflect increased covalent ISGylation of MVB proteins, because USP18, UBE2L6, HERC5/HERC6, and competing ubiquitination pathways can alter the net modification state. Third, studies often measure different endpoints, including extracellular vesicle number, cargo composition, lysosomal degradation, or functional transfer to recipient cells. Finally, cancer cells may rewire endosome–lysosome fusion and secretory trafficking so that an ISG15-rich state promotes, rather than suppresses, pro-metastatic vesicle release. These caveats highlight the need for standardized assays that distinguish free ISG15, substrate ISGylation, vesicle biogenesis, and vesicle function within the same disease context. Exogenous substances or signaling pathways can also affect exosome secretion by modulating ISG15. For example, the peptide WKYMVm activates the formyl peptide receptor 2 (FPR2) pathway, downregulates ISG15 and TFEB expression in mouse bone marrow mesenchymal stem cells (mBMSCs), reduces lysosomal activity, and consequently promotes exosome secretion (64). In dermal mesenchymal stem cells (DMSCs), depletion of peroxiredoxin II (Prx II) reduces JNK activity, which subsequently downregulates the expression of ISGylation-related genes through the miR-221/STAT signaling pathway, leading to intracellular MVB accumulation and reduced exosome secretion (65). ISG15-related pathways influence exosome output through MVB–lysosome trafficking, but the direction of the effect depends on cell type and on whether covalent substrate ISGylation is demonstrated (11, 66).

### 4.3 Mediating signal transduction in endosomes

Endosomes act not only as carriers for endocytosis and cargo sorting but also as important platforms for the activation and regulation of diverse signaling pathways. Emerging evidence suggests that endosomal compartments may function as spatial platforms that support sustained interferon- and ISG15-associated signaling responses. A pioneering study identified a novel mode of type I interferon (IFN-I) signaling: after binding to cell surface receptors, IFN-I is not immediately degraded but is instead “stored” within endosomal compartments, forming so-called “IFN silos” (67). IFN-I within these endosomes is largely functionally dormant because USP18 directly limits type I IFN signaling; in human cells, intracellular ISG15 supports this negative-regulatory function by stabilizing USP18. Accordingly, in cells from ISG15- or USP18-deficient individuals, stored IFN-I can signal continuously from within endosomes, helping to explain the prolonged effects of IFN-I therapy observed *in vivo* and *in vitro* (67). Endosomes may therefore serve as storage and sustained-signaling platforms for IFN responses, with USP18 acting as the direct negative regulator and intracellular ISG15 supporting this checkpoint.

**Figure 4:**
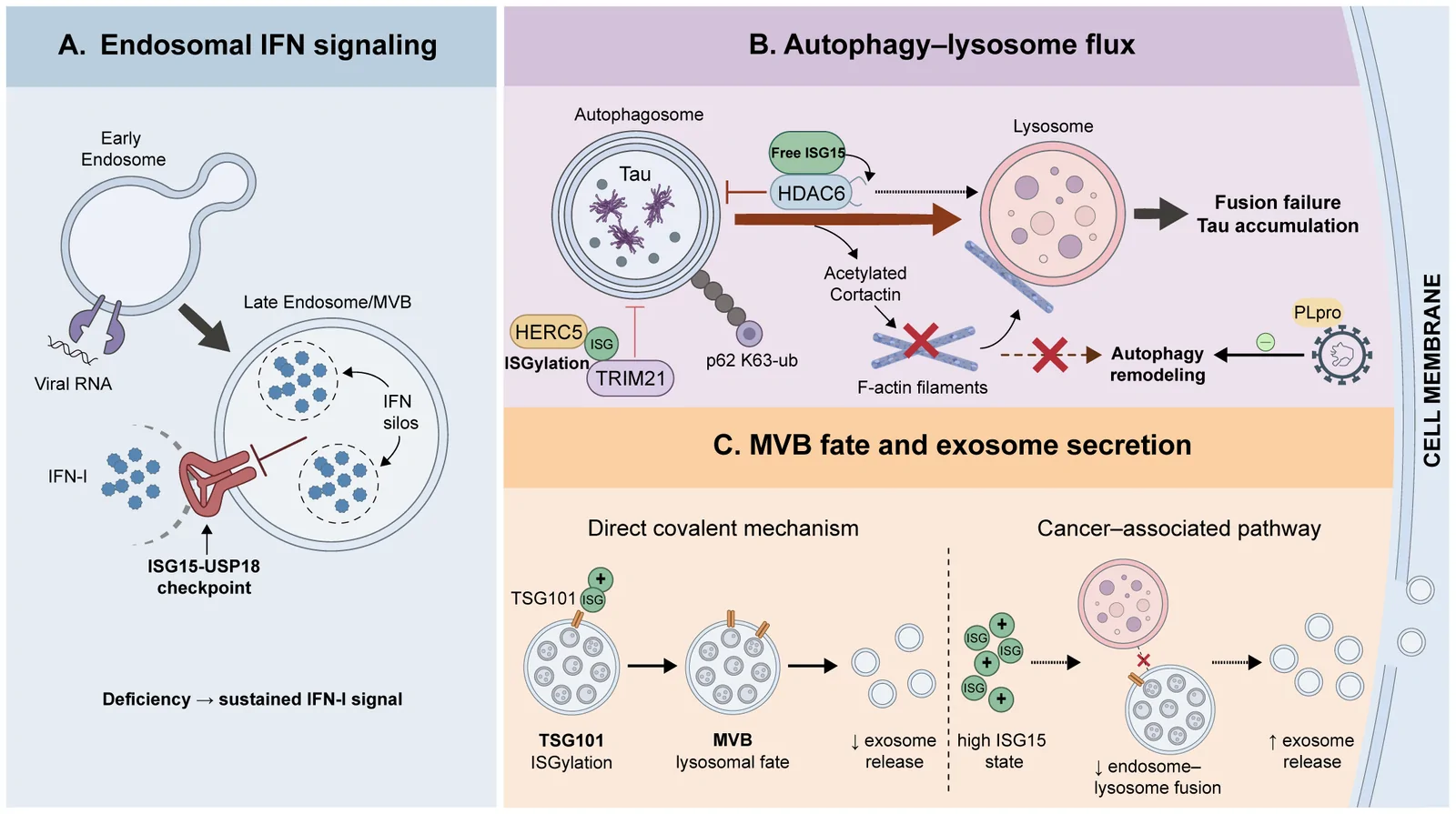
ISGylation remodels endolysosomal flux and exosome secretion. **(A)** Endosomal IFN signaling. The ISG15-USP18 regulatory axis can influence the persistence of IFN signaling from endosomal compartments. **(B)** Autophagy-lysosome flux. Non-covalent ISG15-HDAC6 signaling and p62-related mechanisms affect autophagic flux and lysosomal homeostasis. **(C)** MVB fate and exosome secretion. In canonical IFN-activated settings, ISGylation of TSG101 can redirect MVBs toward lysosomal degradation and suppress exosome release. However, in selected cancer contexts, including ovarian cancer, ISG15-rich states may reduce endosome-lysosome fusion and thereby enhance exosome secretion. Direct substrate-level evidence should be distinguished from ISG15/IFN-associated pathway responses and from cancer-specific rewiring of vesicle trafficking. Solid arrows indicate experimentally supported activation, trafficking, or functional outcomes; inhibitory bars indicate inhibition or blockade; dashed arrows indicate indirect, proposed, or correlative relationships.

Beyond the ISG15–USP18 checkpoint, endocytic trafficking may also induce broader ISG15-associated responses that influence signaling or cargo fate within endosomal compartments. After SARS-CoV-2 S-RBD is internalized by iPSC-derived cardiomyocytes through an ACE2-dependent mechanism, it is transported through RAB5A-positive endosomes to lysosomes, accompanied by the induction of IFN-responsive genes, including ISG15, and increased global protein ISGylation (68). However, the modified substrates and their roles in endosomal trafficking remain unknown. Thus, the ISG15–USP18 checkpoint is supported as a regulator of persistent endosomal IFN signaling, whereas direct covalent remodeling of endosomal trafficking remains poorly defined (**Figure 4**). These context-dependent effects make the endolysosomal system a high-priority but challenging area for disease-oriented validation. Endolysosomal ISG15/ISGylation pathways offer plausible mechanisms for modulating autophagy and exosome release in neurodegeneration, cancer, and tissue repair. Potential readouts include LC3 flux, p62 turnover, HDAC6–cortactin activity, lysosomal fusion, TSG101 ISGylation, extracellular vesicle number, and vesicle cargo. The opposing effects of ISGylation on exosome secretion across different tumor and stromal contexts indicate that biomarker-guided stratification and standardized vesicle assays will be required before this axis can be translated into therapy. The endolysosomal system illustrates why free ISG15, non-covalent ISG15 interactions, and covalent ISGylation should not be considered interchangeable mechanisms. Non-covalent ISG15–HDAC6 binding can impair autophagosome–lysosome fusion, whereas HERC5-dependent TRIM21 ISGylation modifies p62-related autophagic regulation and TSG101 ISGylation can redirect MVB fate toward lysosomal degradation and suppress exosome release. The apparent increase in exosome secretion associated with high ISG15 in ovarian cancer therefore does not constitute direct evidence for an opposite effect of TSG101 ISGylation, because substrate-specific conjugation was not established in that model. Resolving these context-dependent effects will require parallel quantification of free ISG15, global conjugates, and TSG101-specific ISGylation in the same experimental system, together with conjugation-deficient ISG15 rescue, substrate lysine mutants, live-cell measurements of endosome–lysosome fusion, and standardized extracellular-vesicle assays.

## 5 ISGylation, ribosomes, and lipid droplets: regulation of protein translation and lipid metabolism

### 5.1 Co-translational ISGylation associated with ribosomes

Ribosomes are the principal sites of protein synthesis and provide a platform through which ISGylation can intersect with nascent-chain surveillance during interferon-stimulated stress responses, without necessarily implying direct modification of core ribosomal structural proteins. Considerable evidence indicates that ISGylation is not a random cytoplasmic event but is closely coupled to protein translation through a process known as co-translational ISGylation (3). HERC5 and its murine homolog HERC6 are major E3 ligases that mediate ISG15 conjugation in human and mouse systems, respectively. HERC5 physically associates with polyribosomes, allowing it to conjugate ISG15 to nascent polypeptide chains while they are still being synthesized on ribosomes (3, 12). This model of co-translational modification helps explain the broad substrate spectrum of ISGylation, which includes more than one thousand reported candidate substrates, although many modification sites and functional consequences remain unvalidated (12). ISGylation appears to preferentially tag newly synthesized proteins, allowing the cell to rapidly distinguish newly produced proteins, including potential viral proteins, from the pre-existing pool of self proteins during interferon stimulation, such as viral infection (3).

Co-translational ISGylation is considered an important mechanism of host antiviral defense. In IFN-stimulated cells, newly translated viral proteins are likely to represent major targets of ISGylation (3). For instance, ISGylation of the L1 capsid protein of human papillomavirus (HPV) has been shown to dominantly inhibit the infectivity of HPV16 pseudoviruses (3). ISG15 tagging of newly synthesized proteins may facilitate co-translational antigen processing and enhance major histocompatibility complex class I antigen presentation (69). Although an ISG15 fusion protein itself does not directly accelerate degradation, it significantly enhances the presentation efficiency of associated antigenic peptides on major histocompatibility complex class I (69).

Correlative omics studies have linked ISG15 to ribosome- or translation-related signatures in venous thromboembolism and several viral disease datasets, including COVID-19, influenza, and HIV (43, 70, 71). These findings constitute Level 3 association evidence and should not be interpreted as evidence of covalent ISGylation of core ribosomal proteins. Because these datasets derive from different tissues and cellular compositions and often quantify RNA rather than covalent modification, cross-study differences may also reflect cell-type composition and interferon tone rather than ribosome-specific ISGylation. By contrast, direct substrate-level evidence has been established for the translation-associated factor 4EHP, whose ISGylation enhances its cap-structure-binding activity (72). SARS-CoV-2 NSP12 also interacts with eEF1A and alters host mRNA translation, including ISG15 mRNA translation, but this represents viral regulation of the translation machinery rather than evidence of its ISGylation (73). Overall, direct evidence supports HERC5-associated co-translational ISGylation of nascent chains and substrate-level modification of 4EHP, whereas disease-associated ribosomal signatures remain correlative. The key unresolved issue is therefore not whether ISGylation is associated with translating ribosomes, which is well supported, but which nascent-chain modifications are functionally consequential rather than reflecting the broad substrate capture intrinsic to HERC5-associated co-translational ISGylation.

### 5.2 Regulating lipid droplet metabolism and lipolysis

Lipid droplets (LDs) are organelles that store neutral lipids and were long considered inert energy reservoirs. More recent research, however, has shown that LDs play dynamic and active roles in cellular metabolism, signaling, and immune responses. As an important component of the cellular stress response, the ISG15/ISGylation system has been linked to LD metabolism and dynamics, as well as to the function of LDs as immune platforms. In nasopharyngeal carcinoma, expression of UBE2L6, the ISG15-conjugating E2 enzyme, is reduced through promoter hypermethylation and correlates with poor patient prognosis (74). Low UBE2L6 expression leads to marked LD accumulation in nasopharyngeal carcinoma cells and tissues. Mechanistically, UBE2L6 promotes ISGylation of the VCP protein, thereby counteracting the degradation of adipose triglyceride lipase (ATGL). As the key rate-limiting enzyme in lipolysis, destabilization of ATGL impairs lipid breakdown and promotes lipid accumulation (74). This study links ISGylation to dysregulated lipid metabolism in cancer and identifies a novel UBE2L6–ISG15–VCP–ATGL regulatory axis.

The broader ISG15 pathway is also linked to lipid metabolism in the context of infection and immunity. In vaccinia virus (VACV)-infected macrophages, ISG15 is essential for maintaining lipid metabolic homeostasis. ISG15-deficient macrophages exhibit reduced neutral lipid levels and fewer LDs, changes associated with increased lipase abundance rather than altered fatty acid oxidation (FAO) (30). Viral infection further aggravates lipid metabolic disruption in ISG15-deficient cells, potentially in association with increased expression of the metabolic regulators PPARγ and PGC-1α. In VACV-infected macrophages, ISG15 deficiency is associated with reduced neutral-lipid stores and increased lipolysis, supporting a role for the ISG15 pathway in maintaining lipid homeostasis (30). In addition, RNF213, a giant interferon-induced protein, has been identified as a cellular sensor of ISGylated proteins. Following interferon stimulation, RNF213 undergoes ISGylation and oligomerization and localizes to LDs (75). At LDs, RNF213 exerts broad-spectrum antimicrobial activity by directly counteracting infections caused by diverse bacteria and viruses. This finding connects LDs, ISGylation, and direct pathogen clearance, underscoring the importance of LDs as platforms for innate immune defense.

**Figure 5:**
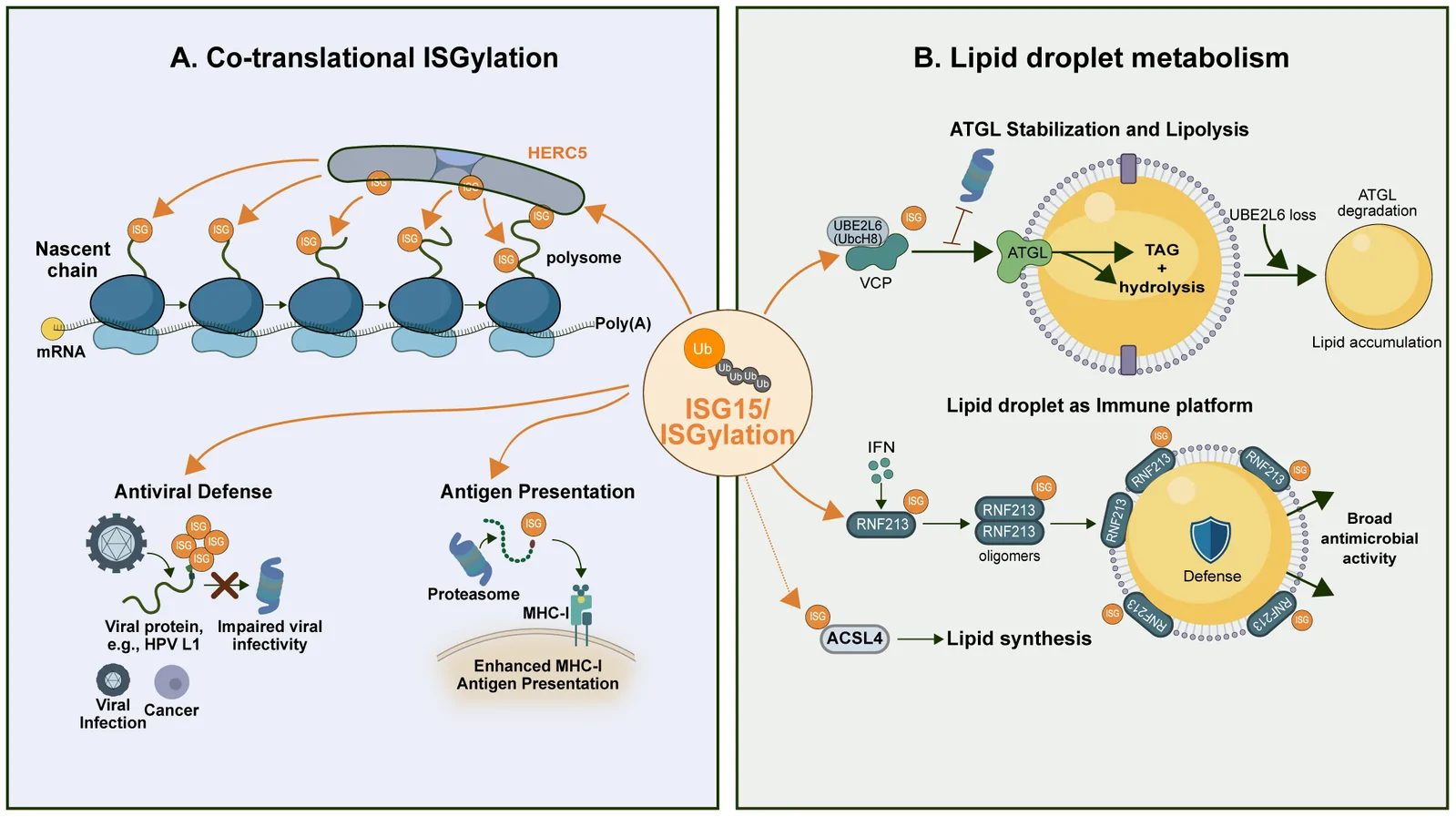
ISGylation links co-translational protein surveillance on ribosomes to lipid droplet metabolism and lipolysis. **(A)** Co-translational ISGylation. HERC5/HERC6-associated nascent-chain modification may restrict viral protein production and modulate antigen presentation. Ribosome-related and translation-associated omics signatures should be interpreted as pathway-level associations unless direct covalent ISGylation of the relevant translation factor, ribosomal component, or nascent substrate is demonstrated. **(B)** Lipid droplet metabolism. UBE2L6-VCP-ATGL, RNF213, and ACSL4-related mechanisms connect ISGylation to lipid storage, lipolysis, antimicrobial defense, and inflammatory metabolic remodeling. Solid arrows indicate experimentally supported activation, trafficking, or functional outcomes; inhibitory bars indicate inhibition or blockade; dashed arrows indicate indirect, proposed, or correlative relationships.

Under other pathological conditions, ISG15-related pathways are also associated with lipid metabolic remodeling. In microglia during sepsis-associated encephalopathy (SAE), upregulation of hexokinase 2 (HK2) is associated with inflammatory responses and abnormal lipid metabolism (76). Knocking down HK2 reduces LD accumulation, a mechanism involving decreased ISG15-mediated ISGylation of acyl-CoA synthetase long-chain family member 4 (ACSL4). This suggests that ISG15-mediated modification of ACSL4 may influence fatty acid activation, lipid synthesis, and lipid storage in inflammatory microglial settings (76). Additionally, an interferon-driven imbalance in the lipid–bile acid metabolic axis has been observed in peripheral blood mononuclear cells from ankylosing spondylitis (AS) patients, with interferon-related genes such as ISG15 associated with pro-oxidant lipid accumulation (77). Accordingly, this finding is best interpreted as a systemic interferon/metabolic association rather than evidence of lipid-droplet-specific ISGylation. Overall, evidence linking ISG15 biology to lipid-droplet regulation varies substantially in mechanistic strength. The most direct evidence supports the UBE2L6–VCP–ATGL axis in nasopharyngeal carcinoma (74), whereas RNF213-associated antimicrobial activity (75) and ACSL4-associated lipid remodeling (76) require further substrate- and site-specific functional validation. Disease-associated lipid signatures, such as those reported in ankylosing spondylitis, remain correlative and should not be interpreted as evidence of lipid-droplet-specific covalent ISGylation (77) (**Figure 5**). Apparent differences in lipid-droplet phenotypes also require context-specific interpretation: ISG15 deficiency is associated with reduced lipid-droplet abundance and increased lipolysis in VACV-infected macrophages (30), whereas UBE2L6 loss promotes lipid accumulation in nasopharyngeal carcinoma (74). These observations arise from different cell types and molecular mechanisms and therefore should not be interpreted as evidence for a universal directional effect of ISG15 on lipid-droplet abundance. Decisive experiments should combine conjugation-deficient ISG15 rescue, substrate-specific lysine mutants, perturbation of UBE2L6 or relevant E3 ligases, and matched measurements of lipolysis, lipid-droplet turnover, and inflammatory or antimicrobial function in disease-relevant models.

## 6 Discussion

For clinical development, organelle-associated ISGylation should be evaluated in relation to disease stratification, biomarker-guided decision-making, and pharmacodynamic monitoring. The key question is which ISG15-related event occurs, in which organelle and cell type, under what disease context, and with what measurable clinical consequence—not simply whether total ISG15 is increased or decreased. Future studies should therefore integrate substrate-specific ISGylation assays with disease-relevant samples, functional organelle readouts, and clinically meaningful endpoints, including disease activity, viral burden, metabolic adaptation, treatment response, relapse risk, toxicity, and survival. Such an approach would help differentiate protective antiviral ISGylation from pathogenic ISG15 programs driven by interferon signaling or organelle stress.

### 6.1 Organelle-resolved ISGylome mapping as a foundation for clinical validation

A major obstacle to translation is the lack of high-confidence, organelle-resolved ISGylomes. Although global ISGylation datasets have expanded the putative substrate repertoire, many identified proteins still require validation of their subcellular localization, modified lysine residues, and functional consequences (3, 12). Future studies should therefore combine subcellular fractionation, proximity labeling, ISG15 remnant profiling, quantitative mass spectrometry, and CRISPR-based perturbation of UBA7, UBE2L6, HERC5/HERC6, and USP18. Rather than being interpreted in isolation, these approaches should be integrated with functional readouts such as mitochondrial respiration, autophagic flux, ER stress signaling, vesicular trafficking, exosome release, lipid droplet dynamics, and live-cell organelle imaging.

These discovery platforms also have technical limitations that should temper mechanistic interpretation. Conventional immunoblotting and immunoprecipitation depend on antibody performance and do not by themselves establish substrate-specific covalent modification. In tryptic remnant proteomics, ubiquitin, NEDD8, and ISG15 can generate the same K-ϵ-GG remnant, meaning that anti-diGly enrichment alone cannot uniquely assign a modification site to ISG15. Genetic controls, such as ISG15- or HERC5/HERC6-deficient systems, quantitative comparison, and orthogonal validation by substrate immunoprecipitation or lysine-site mutagenesis are therefore important for confident site assignment (12). Detection sensitivity is also influenced by modification stoichiometry, interferon exposure, cell type, and subcellular fractionation, so failure to detect a site should not be interpreted as evidence that ISGylation is absent. Transcriptomic datasets are even further removed from covalent modification and should primarily be interpreted as evidence of pathway activation rather than substrate ISGylation.

Patient-derived organoids, primary immune cells, tumor samples, and disease-relevant animal models should be prioritized because ISGylation is strongly influenced by cell type, interferon tone, and stress context (5, 11). A practical discovery-to-translation pipeline could comprise three stages: first, unbiased substrate discovery in organelle-enriched fractions; second, site-level and functional validation using substrate mutants or enzyme perturbation; and third, assay development in annotated patient samples. This workflow would help distinguish organelle-specific ISGylation events from broader interferon-induced bystander changes.

### 6.2 Biomarker development and patient stratification

At the current stage, exploratory biomarker development may be more immediately feasible than direct enzyme inhibition. ISG15, USP18, UBE2L6, HERC5/HERC6, and substrate-specific ISGylation signatures could be evaluated in longitudinal cohorts of viral infection, inflammatory disease, neurodegeneration, and cancer (5, 11, 13). However, biomarker studies should avoid treating *ISG15* mRNA or total ISG15 protein abundance as a direct surrogate for covalent ISGylation. Free intracellular ISG15, extracellular ISG15, total ISGylated proteins, and selected organelle-localized substrates may reflect different biological processes and should ideally be measured in parallel (4, 5).

Potentially informative sample types include peripheral blood mononuclear cells, plasma or serum, extracellular vesicles, tumor tissue, synovial or cartilage specimens, cerebrospinal fluid, patient-derived organoids, and primary immune cells. Screening assays such as immunoblotting, ELISA, or transcriptomic profiling may be useful for detecting broad ISG15 pathway activation, whereas targeted proteomics, immunoprecipitation-mass spectrometry, ISG15 remnant profiling, or substrate-specific assays will be required to confirm covalent ISGylation. This distinction is particularly important because *ISG15* mRNA, free ISG15 protein, total ISGylated proteins, and substrate-specific ISGylation may have different clinical meanings.

For clinical translation, these molecular readouts should be linked to defined endpoints, including disease activity, treatment response, viral load, exosome cargo, metabolic phenotype, interferon toxicity, relapse risk, or survival. In this sense, the value of ISGylation biomarkers may lie not only in diagnosis, but also in patient stratification—identifying patients in whom the ISG15 axis reflects protective antiviral immunity, pathogenic inflammation, metabolic rewiring, or organelle stress.

### 6.3 Therapeutic targeting: opportunities and safety concerns

Therapeutic development should prioritize selectivity, disease context, and safety. Because ISG15 participates in both protective antiviral immunity and pathogenic inflammatory or organelle-stress responses, nonspecific activation or inhibition of the entire ISG15 pathway is unlikely to be clinically optimal (5, 6, 11). Instead, therapeutic strategies should be ranked according to target specificity, disease dependence, and the likelihood of preserving beneficial host immunity.

Among potential therapeutic opportunities, pathogen-encoded de-ISGylating enzymes are attractive because they can be inhibited without directly suppressing the host ISG15-conjugation machinery. SARS-CoV-2 PLpro is the clearest example: in addition to viral polyprotein processing, it removes ubiquitin and ISG15 from host proteins (35, 36). Structure-guided drug development has now produced orally active PLpro inhibitors with antiviral efficacy in mouse infection models, including Jun12682, while more recent optimization has yielded MR1–114 with favorable oral exposure and *in vivo* antiviral activity (78, 79). These advances move PLpro beyond target validation into preclinical lead optimization, although human pharmacokinetics, safety, resistance barriers, and the relative therapeutic contributions of protease inhibition versus deubiquitinating/de-ISGylating inhibition remain unresolved. HCMV pUL50 should be distinguished mechanistically from catalytic viral de-ISGylases: pUL50 suppresses host ISGylation indirectly by promoting RNF170-dependent degradation of UBA7 (41), and therefore requires a different therapeutic strategy.

A second, more disease-specific strategy is to target defined molecular axes within the broader ISG15 system, while distinguishing covalent substrate ISGylation from non-covalent ISG15 interactions. These include the PARP12–ISG15–MFN1/2 axis in osteoarthritis (19), the UBE2L6–VCP–ATGL axis in lipid metabolism (74), the incompletely characterized ISG15–NOX4 pathway in acute kidney injury (28), and the non-covalent ISG15–HDAC6 interaction involved in autophagic-flux impairment (55). These disease-specific axes may offer more precise therapeutic opportunities than global manipulation of the ISG15 pathway, but they require mechanism-appropriate validation, tissue-specific delivery strategies, and biomarkers capable of identifying patients in whom the relevant pathway is active.

By contrast, global manipulation of host ISG15, USP18, UBA7, UBE2L6, or HERC5/HERC6 should be approached with caution. Broad inhibition of ISGylation may reduce pathogenic organelle stress in selected settings, but it could also compromise antiviral defense. Conversely, broad enhancement of ISGylation may strengthen antiviral immunity, while potentially exacerbating interferon-driven inflammation, neurodegenerative stress, or tissue injury (4–6, 11). USP18-targeted approaches are especially complex because USP18 functions both as a de-ISGylating enzyme and as a negative regulator of type I interferon signaling (4, 5).

Future therapeutic development should therefore combine target-selective intervention with biomarker-guided patient stratification. Patients should be classified according to interferon tone, free ISG15 abundance, total ISGylated protein burden, substrate-specific ISGylation, organelle dysfunction, and disease stage. Such stratification will be essential for distinguishing contexts in which ISGylation is protective from those in which it is pathogenic (5, 11).

### 6.4 Tiered roadmap for translational development

Translational readiness should be considered separately from mechanistic evidence strength. A substrate supported by Level 1 evidence may remain clinically remote if substrate-selective modulation is unavailable, the relevant tissue is inaccessible, or pathway manipulation poses substantial immunological risk. Conversely, a pathway-level biomarker may be closer to clinical evaluation if it can be reproducibly measured in accessible patient samples and prospectively associated with treatment response. Translational priorities should therefore be defined jointly by mechanistic confidence, assay feasibility, disease specificity, and therapeutic safety.

In the near term, the most tractable translational opportunity is likely to lie in standardized biomarker and pharmacodynamic validation rather than broad therapeutic manipulation of the ISG15 pathway. Priority should be given to assays that distinguish free ISG15, total ISGylated proteins, and selected substrate-specific modifications, with longitudinal validation against disease activity, treatment response, viral burden, metabolic phenotype, or toxicity. Candidate applications include pharmacodynamic monitoring of host ISGylation during inhibition of pathogen-encoded de-ISGylases (35, 36) and disease-specific evaluation of substrate-level readouts such as MFN1/2 ISGylation in osteoarthritis (19). These approaches require assay standardization, independent patient cohorts, and prospective association with clinically meaningful endpoints before routine clinical application.

Over the medium term, disease- and substrate-selective interventions may become feasible for molecular axes that already have relatively well-defined mechanistic support. Candidate examples include the PARP12–ISG15–MFN1/2 axis in osteoarthritis (19), the UBE2L6–VCP–ATGL axis in nasopharyngeal carcinoma (74), and ISG15-associated NOX4 regulation in acute kidney injury (28). The TSG101–MVB axis may also warrant disease-specific evaluation where altered vesicle trafficking contributes to pathology (62). However, these pathways should not yet be considered clinically actionable. Before therapeutic development, they require independent replication, substrate- or site-specific rescue experiments, demonstration of disease dependence *in vivo*, clinically accessible patient-selection biomarkers, and strategies for tissue- or substrate-selective modulation.

By contrast, several directions remain exploratory because their causal mechanisms, human relevance, or therapeutic selectivity are not yet sufficiently established. These include global manipulation of host ISG15, USP18, UBA7, UBE2L6, or HERC5/HERC6; the human relevance of STING ISGylation (54); broad organelle-resolved ISGylome discovery; and poorly validated ribosome- or lipid-droplet-associated substrates. Global host-pathway intervention is particularly challenging because the same pathway can support antiviral defense while contributing to inflammatory, metabolic, or organelle-stress phenotypes in other contexts. These approaches should therefore remain at the mechanistic discovery and preclinical validation stage until causal substrates, disease-specific therapeutic windows, and safety profiles are established.

Taken together, this tiered framework suggests that progression toward clinical application should depend on the convergence of mechanistic confidence, clinically accessible biomarkers, disease-specific functional relevance, and acceptable safety profiles. On this basis, near-term efforts should emphasize biomarker and pharmacodynamic validation, medium-term development should focus on disease- and substrate-selective interventions, and globally acting or incompletely validated ISG15 pathways should remain exploratory. Mechanistic evidence levels are summarized in **Table 2** and **Supplementary Table 1**, whereas disease- and substrate-specific translational priorities are organized in **Supplementary Table 2**.

### 6.5 Methodological and contextual limitations

Several constraints limit cross-study generalization of organelle-associated ISG15 biology. Increased ISG15 expression or total conjugate abundance does not identify the causal substrate or modification site, and omics-derived associations remain sensitive to assay design, cellular composition, and interferon state. More importantly, ISG15 responses are strongly cell- and disease-context dependent. For example, ISG15 supports mitophagy and metabolic fitness in PaCSCs (18) but is associated with impaired mitophagy in A-T and OA models (15, 16, 19). Similarly, IFN-induced TSG101 ISGylation suppresses exosome release (62), whereas high ISG15 expression in ovarian cancer is associated with increased exosome secretion without establishing the same covalent mechanism (63). Cross-study conclusions should therefore be restricted to the experimental cell type, disease stage, and interferon context unless reproduced in matched systems. Future studies should combine orthogonal substrate validation with controlled interferon conditions, disease-relevant cell models, and longitudinal patient samples to distinguish conserved mechanisms from context-specific responses.

## 7 Conclusions

This review frames ISG15-related biology as a spatially organized component of organelle homeostasis rather than a purely antiviral pathway. Across mitochondria, the ER–Golgi axis, endolysosomal compartments, ribosomes, and lipid droplets, substrate-validated covalent ISGylation has been demonstrated only in selected settings, whereas many additional observations reflect free ISG15, USP18-mediated interferon regulation, or correlative interferon signatures. The strongest mechanistic evidence involves defined substrates such as DRP1, MFN1/2, HK2, PFK1, TSG101, TRIM21 and 4EHP; by contrast, several disease-associated findings remain pathway-level or omics-based. Future work should prioritize organelle-resolved substrate and lysine-site mapping, functional rescue experiments, and validation in disease-relevant patient samples. Clinical development should prioritize substrate-selective or pathogen-selective interventions over global manipulation of the ISG15 pathway, because protective antiviral and pathogenic inflammatory or metabolic effects may coexist. These distinctions are essential for reliable biomarker development, patient stratification, and safe therapeutic targeting.
