# Supplementary material for: Organelle-centered ISG15 biology: distinguishing covalent ISGylation from interferon-associated responses
Source: Front Immunol. 2026 Sep 3;17:1960492. doi: 10.3389/fimmu.2026.1960492 (PMC13584726; doi:10.3389/fimmu.2026.1960492)
Supplement: Supplementary file 1 [file Table1.docx]

**Supplementary Table S1. Organelle-resolved mechanistic evidence for ISG15/ISGylation biology.**

| **Organelle/system** | **Representative mechanism** | **Evidence status** | **Key unresolved issue** | **Reference(s)** |
| --- | --- | --- | --- | --- |
| Mitochondria | DRP1-mediated mitochondrial fission; MFN1/2–PINK1/Parkin mitophagy in OA; ISG15-dependent mitophagy in PaCSCs; mitochondrial metabolic/respiratory remodeling | L1: DRP1, MFN1/2; L2: PaCSC mitophagy; L3: respiratory/metabolic signatures | Why ISG15 promotes or impairs mitophagy and mitochondrial metabolism in different cellular and disease contexts | (18, 19, 23, 26, 27) |
| ER–Golgi axis | TSG101-dependent Golgi trafficking; zebrafish STING ISGylation; ERAD/UPR-associated ISG15 pathways | L1: TSG101; L1 in zebrafish: STING; L2: perturbation-supported ERAD/UPR pathways; L3: association-only mammalian STING/UPR responses | Whether STING ISGylation is conserved and functional in human cells and whether ER proteostasis is directly regulated by ISGylation | (35–42, 45, 51, 54) |
| Endolysosomal system | TSG101-regulated MVB fate; TRIM21/p62 autophagic regulation; non-covalent ISG15–HDAC6 signaling; context-dependent exosome secretion | L1: TSG101, TRIM21; L2: non-covalent HDAC6 and other perturbation-supported pathways; L3: cancer-associated exosome phenotypes without substrate-specific validation | Distinguish covalent ISGylation from free/non-covalent ISG15 effects and explain context-dependent regulation of autophagic flux and exosome secretion | (55–58, 62, 63) |
| Ribosome-associated translation | HERC5/HERC6-associated co-translational ISGylation; 4EHP ISGylation; disease-associated ribosome/translation signatures | L1: 4EHP; L2: co-translational ISGylation with many functionally unresolved substrates; L3: disease-associated omics signatures | Which nascent-chain ISGylation events are functionally consequential | (3, 12, 43, 70–72) |
| Lipid droplets | UBE2L6–VCP–ATGL axis; RNF213-associated antimicrobial LD responses; ACSL4 ISGylation; lipid-associated disease signatures | L1: UBE2L6–VCP–ATGL; L2: RNF213- and ACSL4-related mechanisms; L3: lipid-associated omics signatures | Establish substrate/site-specific causality and explain context-dependent effects on lipid-droplet metabolism | (30, 74–77) |

**Note:** Evidence levels reflect mechanistic strength within the experimental model and species studied and do not imply cross-species conservation or translational readiness. Level assignments refer to the specific mechanisms indicated rather than to the organelle system as a whole.

**Abbreviations:** ACSL4, acyl-CoA synthetase long-chain family member 4; ATGL, adipose triglyceride lipase; DRP1, dynamin-related protein 1; ER, endoplasmic reticulum; ERAD, endoplasmic reticulum-associated degradation; 4EHP, eIF4E homologous protein; HDAC6, histone deacetylase 6; HERC5/HERC6, HECT and RLD domain-containing E3 ubiquitin protein ligases 5 and 6; ISG15, interferon-stimulated gene 15; LD, lipid droplet; MFN, mitofusin; MVB, multivesicular body; p62, sequestosome 1; OA, osteoarthritis; PaCSC, pancreatic cancer stem cell; PINK1, PTEN-induced kinase 1; RNF213, ring finger protein 213; STING, stimulator of interferon genes; TRIM21, tripartite motif-containing protein 21; TSG101, tumor susceptibility gene 101; UBE2L6, ubiquitin-conjugating enzyme E2 L6; UPR, unfolded protein response; VCP, valosin-containing protein.

**Supplementary Table S2. Tiered translational roadmap for organelle-associated ISG15 biology.**

| **Translational tier** | **Representative examples** | **Development focus** | **Key requirement** | **Reference(s)** |
| --- | --- | --- | --- | --- |
| Near-term | ISG15/ISGylation biomarker and pharmacodynamic assays; preclinical PLpro inhibitors; MFN1/2 ISGylation in OA | Analytical and clinical assay validation | Assay specificity and reproducibility; clinically meaningful endpoints; PK/safety evaluation for PLpro inhibitors | (19, 78, 79) |
| Medium-term | PARP12–ISG15–MFN1/2; ISG15–NOX4; UBE2L6–VCP–ATGL; TSG101–MVB | Preclinical development of disease- and substrate-selective interventions | Independent replication; substrate/site-specific validation; in vivo efficacy; patient-selection biomarkers | (19, 28, 62, 74) |
| Exploratory | Global host ISG15/USP18-pathway manipulation; human relevance of STING ISGylation; unresolved ribosome/LD substrates | Mechanistic discovery and preclinical feasibility | Causal substrates; human conservation; therapeutic window and safety | (3, 5, 6, 11, 12, 54, 75, 76) |

**Note:** Translational tiers indicate proposed development priority rather than mechanistic evidence strength. References are representative rather than exhaustive.

**Abbreviations:** ATGL, adipose triglyceride lipase; ISG15, interferon-stimulated gene 15; LD, lipid droplet; MFN, mitofusin; MVB, multivesicular body; NOX4, NADPH oxidase 4; OA, osteoarthritis; PARP12, poly(ADP-ribose) polymerase family member 12; STING, stimulator of interferon genes; TSG101, tumor susceptibility gene 101; UBE2L6, ubiquitin-conjugating enzyme E2 L6; USP18, ubiquitin-specific protease 18; PLpro, papain-like protease; VCP, valosin-containing protein.
